# Supplementary material for: A specimen-level phylogenetic analysis and taxonomic revision of Diplodocidae (Dinosauria, Sauropoda)
Source: PeerJ. 2015 Apr 7;3:e857. doi: 10.7717/peerj.857 (PMC4393826; doi:10.7717/peerj.857)
Supplement: Supplemental Information 1 [file peerj-03-857-s001.pdf]

Table 3: Definitions of positional terms for vertebrae.

Vertebrae	Subdivision	Definition	Example *Apatosaurus louisae*
Cervical	Anterior	The division is made numerically	CV 1-5
	Mid-cervical		CV 6-10
	Posterior		CV 11-15
Dorsal	Anterior	Parapophysis still touching centrum	DV 1-2
	Mid-dorsals	Numerical subdivision	DV 3-6
	Posterior		DV 7-10
Caudal	Anterior-most	With transverse processes extending onto neural arch	Cd 1-6
	Anterior	With normal transverse process	Cd 7-14
	Mid-caudal	without transverse processes, but still well-developed neural spine	Cd 15-28
	Posterior	Postzygapophyses reduced	Cd 29-42
	Distal	Neural arch reduced	Cd 43-82
