# Supplementary material for: A specimen-level phylogenetic analysis and taxonomic revision of Diplodocidae (Dinosauria, Sauropoda)
Source: PeerJ. 2015 Apr 7;3:e857. doi: 10.7717/peerj.857 (PMC4393826; doi:10.7717/peerj.857)
Supplement: Supplemental Information 5 [file peerj-03-857-s005.docx]

**Supplementary data 1**

Apatosaurinae, weighted pairwise dissimilarity values within and between the species accepted herein

|  | *Apatosaurus ajax* | *Apatosaurus louisae* | NSMT-PV 20375 + “*Atlantosaurus*” *immanis* YPM 1840 | *Brontosaurus excelsus* | *“Eobrontosaurus” yahnahpin* | *“Elosaurus” parvus* |
| --- | --- | --- | --- | --- | --- | --- |
| *Apatosaurus ajax* | NA | 0.21 | 0.21 | 0.18 | 0.26 | 0.24 |
| *Apatosaurus louisae* |  | 0.12 | 0.31 | 0.24 | 0.25 | 0.31 |
| NSMT-PV 20375 + “*Atlantosaurus*” *immanis* YPM 1840 |  |  | 0.20 | 0.23 | 0.26 | 0.28 |
| *Brontosaurus excelsus* |  |  |  | 0.14 | 0.20 | 0.21 |
| *“Eobrontosaurus” yahnahpin* |  |  |  |  | NA | 0.23 |
| *“Elosaurus” parvus* |  |  |  |  |  | 0.23 |

Apatosaurinae, weighted pairwise dissimilarity values within and between the genera accepted herein

|  | *Apatosaurus* | Apatosaurinae ngen | *Brontosaurus* |
| --- | --- | --- | --- |
| *Apatosaurus* | 0.18 | 0.27 | 0.26 |
| Apatosaurinae ngen |  | 0.20 | 0.26 |
| *Brontosaurus* |  |  | 0.21 |

Diplodocinae, weighted pairwise dissimilarity values within and between the species accepted herein

|  | *Barosaurus lentus* | *Diplodocus carnegii* | *Diplodocus hallorum* | *Galeamopus hayi* | *Kaatedocus siberi* | *Leinkupal laticaudata* | *Supersaurus vivianae* | *Supersaurus lourinhanensis* | *Tornieria africana* | Ngen nsp1 |
| --- | --- | --- | --- | --- | --- | --- | --- | --- | --- | --- |
| *B. lentus* | 0.12 | 0.20 | 0.20 | 0.23 | 0.25 | 0.18 | 0.30 | 0.28 | 0.28 | 0.38 |
| *D. carnegii* |  | 0.06 | 0.12 | 0.21 | 0.31 | 0.22 | 0.35 | 0.28 | 0.24 | 0.37 |
| *D. hallorum* |  |  | 0.05 | 0.24 | 0.29 | 0.23 | 0.36 | 0.31 | 0.24 | 0.41 |
| *G. hayi* |  |  |  | NA | 0.31 | 0.25 | 0.26 | 0.25 | 0.27 | 0.32 |
| *K. siberi* |  |  |  |  | 0.04 | NA | 0.29 | 0.22 | 0.33 | 0.00 |
| *L. laticaudata* |  |  |  |  |  | NA | 0.28 | 0.00 | 0.22 | 0.33 |
| *S. vivianae* |  |  |  |  |  |  | 0.07 | 0.20 | 0.29 | 0.28 |
| *S. lourinhanensis* |  |  |  |  |  |  |  | NA | 0.33 | 0.41 |
| *T. africana* |  |  |  |  |  |  |  |  | 0.03 | 0.31 |
| Ngen nsp1 |  |  |  |  |  |  |  |  |  | 0.11 |

Diplodocinae, weighted pairwise dissimilarity values within and between the genera accepted herein

|  | *Barosaurus* | *Diplodocus* | *Galeamopus* | *Kaatedocus* | *Leinkupal* | *Supersaurus* | *Tornieria* | Ngen |
| --- | --- | --- | --- | --- | --- | --- | --- | --- |
| *Barosaurus* | 0.12 | 0.20 | 0.27 | 0.25 | 0.18 | 0.30 | 0.28 | 0.38 |
| *Diplodocus* |  | 0.09 | 0.25 | 0.30 | 0.23 | 0.34 | 0.24 | 0.40 |
| *Galeamopus* |  |  | 0.18 | 0.30 | 0.25 | 0.32 | 0.27 | 0.31 |
| *Kaatedocus* |  |  |  | 0.04 | NA | 0.26 | 0.33 | 0.00 |
| *Leinkupal* |  |  |  |  | NA | 0.26 | 0.22 | 0.33 |
| *Supersaurus* |  |  |  |  |  | 0.14 | 0.30 | 0.30 |
| *Tornieria* |  |  |  |  |  |  | 0.03 | 0.31 |
| Ngen |  |  |  |  |  |  |  | 0.11 |
