# Supplementary material for: A specimen-level phylogenetic analysis and taxonomic revision of Diplodocidae (Dinosauria, Sauropoda)
Source: PeerJ. 2015 Apr 7;3:e857. doi: 10.7717/peerj.857 (PMC4393826; doi:10.7717/peerj.857)
Supplement: Supplementary file 7 [file peerj-03-857-s008.odt]

### Supplementary Data 4

### Synapomorphies implied weighting analysis

Please note that character numbering starts with 0, not 1.

### R_iw

Tree 60001 

Shunosaurus lii 

  No autapomorphies 

Spinophorosaurus nigerensis 

  Char. 0: 1 --> 2 

  Char. 25: 0 --> 1 

  Char. 28: 0 --> 1 

  Char. 63: 0 --> 1 

  Char. 65: 0 --> 1 

  Char. 90: 0 --> 1 

  Char. 97: 0 --> 1 

  Char. 160: 0 --> 1 

  Char. 165: 0 --> 1 

  Char. 179: 0 --> 1 

  Char. 180: 0 --> 1 

  Char. 181: 0 --> 1 

  Char. 186: 0 --> 1 

  Char. 189: 0 --> 1 

  Char. 195: 0 --> 1 

  Char. 199: 0 --> 1 

  Char. 202: 0 --> 1 

  Char. 273: 0 --> 1 

  Char. 298: 1 --> 0 

  Char. 326: 0 --> 1 

  Char. 331: 0 --> 1 

  Char. 333: 0 --> 2 

  Char. 366: 0 --> 1 

  Char. 379: 1 --> 0 

Omeisaurus 

  Char. 72: 0 --> 1 

  Char. 76: 0 --> 1 

  Char. 165: 0 --> 2 

  Char. 175: 0 --> 1 

  Char. 197: 0 --> 1 

  Char. 278: 1 --> 0 

  Char. 284: 0 --> 1 

  Char. 327: 0 --> 1 

  Char. 342: 0 --> 1 

  Char. 358: 0 --> 1 

  Char. 372: 0 --> 1 

  Char. 386: 1 --> 2 

  Char. 392: 0 --> 1 

  Char. 413: 0 --> 1 

  Char. 475: 1 --> 0 

Mamenchisaurus 

  Char. 57: 0 --> 1 

  Char. 62: 1 --> 0 

  Char. 73: 0 --> 1 

  Char. 125: 0 --> 1 

  Char. 129: 01 --> 2 

  Char. 170: 2 --> 1 

  Char. 206: 0 --> 1 

  Char. 227: 01 --> 2 

  Char. 253: 0 --> 1 

  Char. 272: 0 --> 1 

  Char. 294: 0 --> 1 

  Char. 303: 0 --> 3 

  Char. 357: 1 --> 0 

  Char. 369: 0 --> 1 

  Char. 398: 0 --> 1 

  Char. 425: 0 --> 1 

  Char. 465: 1 --> 0 

  Char. 474: 0 --> 1 

Jobaria tiguidensis 

  Char. 122: 1 --> 0 

  Char. 134: 1 --> 0 

  Char. 140: 0 --> 1 

  Char. 152: 0 --> 2 

  Char. 156: 1 --> 0 

  Char. 159: 0 --> 1 

  Char. 180: 0 --> 1 

  Char. 183: 0 --> 1 

  Char. 184: 1 --> 0 

  Char. 189: 0 --> 1 

  Char. 197: 0 --> 1 

  Char. 212: 0 --> 1 

  Char. 223: 1 --> 0 

  Char. 251: 1 --> 0 

  Char. 265: 1 --> 0 

  Char. 269: 2 --> 0 

  Char. 275: 1 --> 0 

  Char. 278: 1 --> 0 

  Char. 352: 0 --> 1 

  Char. 392: 0 --> 1 

  Char. 407: 1 --> 0 

  Char. 432: 0 --> 1 

  Char. 435: 0 --> 1 

  Char. 444: 0 --> 1 

Turiasaurus riodevensis 

  No autapomorphies 

Losillasaurus giganteus type 

  Char. 260: 0 --> 1 

  Char. 261: 0 --> 1 

  Char. 309: 0 --> 1 

  Char. 386: 1 --> 0 

Camarasaurus 

  Char. 89: 0 --> 1 

  Char. 260: 0 --> 1 

  Char. 351: 0 --> 1 

  Char. 386: 1 --> 2 

  Char. 432: 0 --> 1 

  Char. 437: 0 --> 1 

Amphicoelias latus AMNH 5765 

  No autapomorphies 

Apatosaurus grandis YPM 1901 

  Char. 340: 0 --> 1 

  Char. 344: 0 --> 1 

  Char. 348: 0 --> 1 

  Char. 352: 0 --> 1 

  Char. 361: 2 --> 1 

  Char. 363: 0 --> 1 

  Char. 373: 2 --> 1 

  Char. 429: 1 --> 2 

  Char. 450: 0 --> 1 

  Char. 458: 0 --> 1 

  Char. 465: 1 --> 0 

Dystrophaeus viaemalae USNM 2364 

  Char. 369: 0 --> 1 

Lourinhasaurus alenquerensis lectotype 

  Char. 303: 0 --> 1 

  Char. 305: 0 --> 1 

  Char. 369: 0 --> 1 

  Char. 379: 1 --> 0 

  Char. 392: 0 --> 1 

  Char. 409: 0 --> 1 

  Char. 420: 0 --> 1 

  Char. 423: 1 --> 0 

  Char. 425: 0 --> 1 

Giraffatitan brancai 

  Char. 62: 1 --> 0 

  Char. 82: 1 --> 0 

  Char. 117: 0 --> 1 

  Char. 165: 0 --> 2 

  Char. 233: 0 --> 1 

  Char. 260: 0 --> 1 

  Char. 261: 0 --> 1 

  Char. 289: 0 --> 1 

  Char. 303: 0 --> 1 

  Char. 305: 0 --> 1 

  Char. 312: 0 --> 1 

  Char. 372: 0 --> 1 

  Char. 382: 1 --> 0 

  Char. 385: 1 --> 0 

SMA 0009 

  Char. 132: 0 --> 1 

  Char. 157: 0 --> 1 

  Char. 167: 1 --> 0 

  Char. 172: 0 --> 1 

  Char. 199: 0 --> 1 

  Char. 217: 0 --> 1 

  Char. 238: 0 --> 1 

  Char. 254: 0 --> 1 

  Char. 261: 0 --> 1 

  Char. 265: 1 --> 0 

  Char. 278: 1 --> 0 

  Char. 284: 0 --> 2 

  Char. 294: 0 --> 2 

  Char. 303: 0 --> 1 

  Char. 320: 0 --> 1 

  Char. 329: 0 --> 1 

  Char. 333: 0 --> 1 

  Char. 341: 0 --> 1 

  Char. 363: 0 --> 1 

  Char. 371: 0 --> 1 

  Char. 393: 1 --> 0 

  Char. 395: 0 --> 1 

  Char. 412: 0 --> 1 

  Char. 431: 1 --> 0 

  Char. 433: 0 --> 1 

  Char. 439: 0 --> 1 

  Char. 465: 1 --> 0 

  Char. 475: 1 --> 0 

Brachiosaurus altithorax 

  Char. 25: 0 --> 1 

  Char. 30: 0 --> 1 

  Char. 326: 0 --> 1 

  Char. 404: 0 --> 1 

  Char. 407: 0 --> 1 

  Char. 412: 0 --> 1 

  Char. 431: 1 --> 0 

  Char. 433: 0 --> 1 

Australodocus bohetii type 

  Char. 129: 1 --> 2 

  Char. 166: 0 --> 1 

  Char. 170: 2 --> 1 

  Char. 173: 0 --> 1 

  Char. 178: 1 --> 0 

  Char. 188: 1 --> 0 

  Char. 217: 1 --> 0 

Ligabuesaurus leanzai 

  Char. 127: 1 --> 2 

  Char. 140: 0 --> 1 

  Char. 197: 0 --> 1 

  Char. 242: 0 --> 1 

  Char. 363: 0 --> 1 

  Char. 369: 0 --> 1 

Isisaurus colberti 

  Char. 191: 1 --> 0 

  Char. 235: 0 --> 1 

  Char. 358: 0 --> 1 

Haplocanthosaurus priscus 

  Char. 158: 0 --> 1 

  Char. 166: 0 --> 1 

  Char. 173: 0 --> 1 

  Char. 176: 0 --> 1 

  Char. 184: 1 --> 0 

  Char. 186: 0 --> 1 

  Char. 200: 0 --> 1 

  Char. 223: 1 --> 0 

  Char. 227: 1 --> 0 

  Char. 236: 0 --> 1 

  Char. 241: 0 --> 1 

  Char. 246: 0 --> 1 

  Char. 263: 0 --> 1 

  Char. 287: 0 --> 1 

  Char. 296: 0 --> 1 

  Char. 323: 0 --> 1 

  Char. 348: 0 --> 1 

  Char. 372: 0 --> 1 

  Char. 411: 0 --> 1 

  Char. 420: 0 --> 1 

Limaysaurus tessonei 

  Char. 186: 0 --> 1 

  Char. 296: 0 --> 1 

  Char. 305: 0 --> 1 

  Char. 349: 0 --> 1 

Cathartesaura anaerobica 

  Char. 198: 0 --> 1 

  Char. 320: 0 --> 1 

  Char. 338: 1 --> 0 

  Char. 342: 0 --> 1 

Zapalasaurus bonapartei 

  Char. 308: 0 --> 1 

  Char. 390: 0 --> 1 

Nigersaurus taqueti 

  Char. 106: 0 --> 1 

  Char. 111: 0 --> 1 

  Char. 113: 1 --> 0 

  Char. 165: 0 --> 1 

  Char. 170: 1 --> 2 

  Char. 182: 0 --> 1 

  Char. 184: 1 --> 2 

  Char. 213: 1 --> 0 

  Char. 237: 0 --> 1 

  Char. 257: 0 --> 1 

  Char. 327: 0 --> 1 

Demandasaurus darwini 

  Char. 113: 1 --> 2 

  Char. 118: 1 --> 0 

  Char. 193: 0 --> 1 

  Char. 272: 0 --> 1 

  Char. 281: 1 --> 2 

  Char. 324: 1 --> 0 

  Char. 326: 0 --> 1 

  Char. 329: 0 --> 1 

  Char. 332: 0 --> 1 

  Char. 333: 2 --> 3 

  Char. 429: 1 --> 2 

Amphicoelias altus AMNH 5764 

  Char. 255: 0 --> 1 

  Char. 264: 0 --> 1 

  Char. 267: 1 --> 0 

  Char. 274: 1 --> 0 

  Char. 426: 12 --> 0 

Dicraeosaurus hansemanni 

  Char. 32: 0 --> 1 

  Char. 46: 0 --> 1 

  Char. 59: 1 --> 0 

  Char. 132: 0 --> 1 

  Char. 137: 0 --> 1 

  Char. 170: 1 --> 0 

  Char. 181: 1 --> 0 

  Char. 211: 0 --> 1 

  Char. 216: 0 --> 1 

  Char. 429: 1 --> 0 

  Char. 443: 1 --> 0 

Brachytrachelopan mesai 

  Char. 197: 0 --> 1 

  Char. 213: 1 --> 0 

  Char. 407: 0 --> 1 

Amargasaurus cazaui 

  Char. 56: 0 --> 1 

  Char. 62: 1 --> 0 

  Char. 80: 0 --> 1 

  Char. 96: 0 --> 1 

  Char. 183: 0 --> 1 

  Char. 186: 0 --> 1 

  Char. 188: 1 --> 0 

  Char. 206: 1 --> 0 

  Char. 209: 1 --> 0 

  Char. 242: 0 --> 1 

  Char. 243: 1 --> 0 

  Char. 257: 0 --> 1 

  Char. 303: 1 --> 0 

  Char. 381: 0 --> 1 

  Char. 386: 1 --> 0 

  Char. 387: 1 --> 0 

  Char. 390: 0 --> 1 

  Char. 411: 0 --> 1 

  Char. 431: 1 --> 0 

  Char. 432: 0 --> 1 

Suuwassea emilieae ANS 21122 

  No autapomorphies 

Apatosaurus ajax YPM 1860 

  Char. 132: 1 --> 0 

  Char. 163: 0 --> 1 

  Char. 167: 1 --> 0 

  Char. 168: 0 --> 1 

  Char. 186: 0 --> 1 

  Char. 196: 1 --> 0 

  Char. 197: 0 --> 1 

  Char. 207: 1 --> 0 

  Char. 237: 0 --> 1 

  Char. 257: 1 --> 0 

  Char. 270: 1 --> 0 

  Char. 278: 0 --> 1 

  Char. 291: 0 --> 1 

  Char. 367: 1 --> 0 

Apatosaurus laticollis YPM 1861 

  Char. 127: 1 --> 2 

  Char. 176: 0 --> 1 

  Char. 197: 0 --> 1 

  Char. 200: 1 --> 0 

Brontosaurus excelsus YPM 1980 

  Char. 237: 1 --> 0 

  Char. 354: 1 --> 0 

Brontosaurus amplus YPM 1981 

  Char. 260: 1 --> 0 

  Char. 353: 1 --> 0 

  Char. 373: 1 --> 0 

  Char. 374: 1 --> 0 

  Char. 375: 0 --> 1 

  Char. 411: 0 --> 1 

  Char. 421: 1 --> 0 

  Char. 435: 0 --> 1 

Apatosaurus louisae type 

  No autapomorphies 

Atlantosaurus immanis YPM 1840 

  Char. 141: 0 --> 1 

  Char. 217: 0 --> 1 

Elosaurus parvus CM 566 

  Char. 134: 1 --> 0 

  Char. 137: 1 --> 0 

  Char. 226: 1 --> 0 

  Char. 251: 1 --> 0 

  Char. 253: 0 --> 1 

  Char. 257: 1 --> 0 

  Char. 260: 1 --> 0 

  Char. 365: 1 --> 0 

  Char. 385: 1 --> 0 

  Char. 436: 0 --> 1 

  Char. 447: 0 --> 1 

UW 15556 

  Char. 182: 0 --> 1 

  Char. 201: 0 --> 1 

  Char. 208: 1 --> 0 

  Char. 241: 0 --> 1 

  Char. 304: 1 --> 0 

  Char. 388: 1 --> 0 

Apatosaurus minimus AMNH 675 

  Char. 281: 0 --> 1 

  Char. 285: 0 --> 2 

  Char. 287: 0 --> 1 

  Char. 289: 0 --> 1 

  Char. 406: 0 --> 1 

  Char. 407: 0 --> 1 

  Char. 414: 0 --> 1 

  Char. 419: 0 --> 1 

Eobrontosaurus yahnahpin Tate-001

  Char. 127: 1 --> 2 

  Char. 158: 1 --> 0 

  Char. 217: 0 --> 1 

  Char. 244: 1 --> 0 

  Char. 264: 0 --> 1 

  Char. 320: 1 --> 0 

  Char. 338: 0 --> 1 

  Char. 371: 1 --> 0 

  Char. 392: 0 --> 1 

  Char. 393: 1 --> 0 

  Char. 398: 0 --> 1 

NSMT-PV 20375 

  Char. 182: 0 --> 1 

  Char. 242: 0 --> 1 

  Char. 417: 0 --> 1 

  Char. 419: 1 --> 0 

  Char. 430: 0 --> 1 

  Char. 435: 0 --> 1 

LACM 52844 

  Char. 133: 1 --> 0 

  Char. 157: 0 --> 1 

  Char. 295: 1 --> 0 

  Char. 303: 1 --> 0 

  Char. 331: 0 --> 1 

  Char. 381: 0 --> 1 

  Char. 432: 0 --> 1 

AMNH 460 

  Char. 141: 0 --> 1 

  Char. 267: 1 --> 0 

  Char. 270: 0 --> 1 

  Char. 407: 0 --> 1 

  Char. 411: 0 --> 1 

CM 3378 

  Char. 128: 0 --> 1 

  Char. 158: 0 --> 1 

  Char. 173: 0 --> 1 

  Char. 193: 0 --> 1 

  Char. 221: 0 --> 1 

  Char. 223: 0 --> 2 

  Char. 226: 0 --> 1 

  Char. 251: 0 --> 1 

  Char. 257: 0 --> 1 

  Char. 299: 0 --> 3 

  Char. 305: 0 --> 1 

  Char. 319: 0 --> 1 

  Char. 322: 0 --> 1 

  Char. 329: 0 --> 1 

  Char. 332: 0 --> 1 

  Char. 338: 0 --> 1 

  Char. 343: 0 --> 1 

  Char. 346: 0 --> 1 

  Char. 347: 0 --> 1 

  Char. 357: 1 --> 0 

FMNH P25112 

  Char. 225: 1 --> 0 

  Char. 258: 0 --> 1 

  Char. 269: 0 --> 1 

  Char. 295: 1 --> 0 

  Char. 308: 0 --> 1 

  Char. 323: 0 --> 1 

  Char. 411: 0 --> 1 

  Char. 415: 1 --> 0 

  Char. 419: 1 --> 0 

  Char. 429: 0 --> 2 

  Char. 430: 0 --> 1 

ML 418 

  Char. 178: 1 --> 0 

SMA 0087 

  No autapomorphies 

Supersaurus vivianae BYU  

  Char. 258: 1 --> 0 

  Char. 274: 1 --> 0 

WDC DMJ-021 

  Char. 165: 1 --> 2 

  Char. 172: 0 --> 1 

  Char. 174: 0 --> 1 

  Char. 257: 1 --> 2 

Dinheirosaurus lourinhanensis ML 414

  Char. 125: 1 --> 0 

  Char. 138: 0 --> 1 

  Char. 187: 0 --> 1 

  Char. 229: 0 --> 1 

  Char. 280: 0 --> 1 

  Char. 304: 1 --> 0 

Kaatedocus siberi SMA 0004 

  Char. 85: 0 --> 1 

  Char. 166: 0 --> 1 

Tornieria africana holotype 

  Char. 330: 0 --> 1 

Tornieria africana skeleton k 

  No autapomorphies 

Diplodocus longus YPM 1920 

  Char. 305: 0 --> 1 

  Char. 329: 0 --> 1 

  Char. 331: 0 --> 1 

  Char. 333: 0 --> 2 

  Char. 334: 0 --> 1 

  Char. 338: 0 --> 1 

  Char. 343: 0 --> 1 

Diplodocus lacustris YPM 1922 

  Char. 116: 0 --> 1 

  Char. 118: 0 --> 1 

  Char. 119: 0 --> 1 

  Char. 120: 0 --> 1 

  Char. 124: 0 --> 1 

Diplodocus carnegii CM 84 

  Char. 407: 1 --> 0 

  Char. 411: 1 --> 0 

Diplodocus carnegii CM 94 

  Char. 134: 0 --> 1 

  Char. 186: 0 --> 1 

  Char. 200: 0 --> 1 

  Char. 237: 1 --> 0 

  Char. 240: 1 --> 0 

  Char. 353: 0 --> 1 

  Char. 365: 0 --> 1 

  Char. 366: 0 --> 1 

AMNH 223 

  Char. 277: 1 --> 0 

  Char. 337: 0 --> 1 

  Char. 358: 0 --> 1 

  Char. 368: 0 --> 1 

  Char. 411: 1 --> 0 

USNM 10865 

  Char. 340: 0 --> 1 

DMNS 1494 

  Char. 420: 1 --> 0 

  Char. 421: 0 --> 1 

  Char. 447: 1 --> 0 

AMNH 969 

  Char. 82: 1 --> 0 

  Char. 111: 0 --> 1 

  Char. 149: 0 --> 1 

CM 11161 

  Char. 0: 1 --> 0 

  Char. 1: 0 --> 1 

  Char. 2: 0 --> 1 

  Char. 3: 1 --> 2 

  Char. 4: 0 --> 2 

  Char. 7: 0 --> 1 

  Char. 12: 0 --> 1 

  Char. 13: 0 --> 1 

  Char. 14: 0 --> 1 

  Char. 16: 0 --> 1 

  Char. 17: 0 --> 1 

  Char. 19: 0 --> 1 

  Char. 20: 0 --> 1 

  Char. 22: 0 --> 1 

  Char. 23: 0 --> 1 

  Char. 24: 0 --> 1 

  Char. 26: 0 --> 1 

  Char. 30: 0 --> 1 

  Char. 31: 1 --> 0 

  Char. 33: 0 --> 1 

  Char. 34: 0 --> 1 

  Char. 36: 0 --> 1 

  Char. 37: 0 --> 1 

  Char. 39: 0 --> 1 

  Char. 40: 0 --> 1 

  Char. 43: 0 --> 1 

  Char. 44: 0 --> 1 

  Char. 45: 0 --> 1 

  Char. 48: 0 --> 1 

  Char. 52: 0 --> 1 

  Char. 53: 0 --> 1 

  Char. 54: 0 --> 1 

  Char. 55: 0 --> 1 

  Char. 59: 0 --> 1 

  Char. 62: 0 --> 1 

  Char. 67: 0 --> 1 

  Char. 72: 0 --> 1 

  Char. 76: 0 --> 1 

  Char. 78: 0 --> 1 

  Char. 81: 0 --> 1 

  Char. 82: 0 --> 1 

  Char. 84: 0 --> 2 

  Char. 86: 0 --> 1 

  Char. 88: 0 --> 1 

  Char. 90: 0 --> 1 

  Char. 91: 0 --> 1 

  Char. 92: 0 --> 1 

  Char. 93: 0 --> 1 

  Char. 98: 0 --> 1 

  Char. 101: 0 --> 1 

  Char. 102: 0 --> 1 

  Char. 103: 0 --> 1 

  Char. 107: 0 --> 1 

  Char. 108: 0 --> 1 

  Char. 109: 0 --> 1 

  Char. 110: 0 --> 1 

  Char. 112: 0 --> 2 

  Char. 113: 0 --> 1 

  Char. 115: 0 --> 1 

  Char. 116: 0 --> 1 

  Char. 118: 0 --> 1 

  Char. 119: 0 --> 1 

  Char. 120: 0 --> 1 

  Char. 121: 0 --> 1 

  Char. 124: 0 --> 1 

CM 3452 

  Char. 34: 0 --> 1 

  Char. 43: 0 --> 1 

  Char. 88: 1 --> 0 

  Char. 134: 0 --> 1 

  Char. 173: 0 --> 1 

USNM 2672 

  Char. 0: 1 --> 0 

  Char. 1: 0 --> 1 

  Char. 2: 0 --> 1 

  Char. 3: 1 --> 2 

  Char. 4: 0 --> 2 

  Char. 7: 0 --> 1 

  Char. 12: 0 --> 1 

  Char. 13: 0 --> 1 

  Char. 14: 0 --> 1 

  Char. 16: 0 --> 1 

  Char. 17: 0 --> 1 

  Char. 19: 0 --> 1 

  Char. 20: 0 --> 1 

  Char. 22: 0 --> 1 

  Char. 23: 0 --> 1 

  Char. 24: 0 --> 1 

  Char. 26: 0 --> 1 

  Char. 30: 0 --> 1 

  Char. 31: 1 --> 0 

  Char. 33: 0 --> 1 

  Char. 35: 0 --> 1 

  Char. 36: 0 --> 1 

  Char. 37: 0 --> 1 

  Char. 39: 0 --> 1 

  Char. 40: 0 --> 1 

  Char. 41: 0 --> 1 

  Char. 43: 0 --> 1 

  Char. 44: 0 --> 1 

  Char. 45: 0 --> 1 

  Char. 48: 0 --> 1 

  Char. 52: 0 --> 1 

  Char. 53: 0 --> 1 

  Char. 54: 0 --> 1 

  Char. 55: 0 --> 1 

  Char. 59: 0 --> 1 

  Char. 62: 0 --> 1 

  Char. 67: 0 --> 1 

  Char. 72: 0 --> 1 

  Char. 74: 0 --> 1 

  Char. 81: 0 --> 1 

  Char. 84: 0 --> 2 

  Char. 86: 0 --> 1 

  Char. 87: 0 --> 1 

  Char. 88: 0 --> 1 

  Char. 91: 0 --> 1 

  Char. 92: 0 --> 1 

  Char. 93: 0 --> 1 

  Char. 99: 1 --> 0 

  Char. 101: 0 --> 1 

  Char. 103: 0 --> 1 

  Char. 107: 0 --> 1 

  Char. 108: 0 --> 1 

  Char. 109: 0 --> 1 

  Char. 110: 0 --> 1 

  Char. 112: 0 --> 2 

  Char. 113: 0 --> 1 

  Char. 115: 0 --> 1 

  Char. 116: 0 --> 1 

  Char. 118: 0 --> 1 

  Char. 119: 0 --> 1 

  Char. 120: 0 --> 1 

  Char. 121: 0 --> 1 

  Char. 124: 0 --> 1 

  Char. 143: 0 --> 1 

  Char. 144: 0 --> 1 

WDC-FS001A 

  Char. 356: 0 --> 1 

  Char. 450: 0 --> 1 

  Char. 467: 0 --> 1 

Galeamopus hayi HMNS 175 

  Char. 133: 1 --> 0 

  Char. 138: 0 --> 1 

Galeamopus sp. SMA 0011 

  Char. 82: 1 --> 0 

  Char. 87: 1 --> 0 

  Char. 139: 1 --> 2 

  Char. 153: 1 --> 2 

  Char. 161: 0 --> 1 

  Char. 181: 1 --> 0 

  Char. 185: 0 --> 1 

  Char. 190: 0 --> 1 

  Char. 225: 1 --> 0 

  Char. 246: 0 --> 1 

  Char. 278: 1 --> 0 

  Char. 282: 1 --> 0 

  Char. 379: 1 --> 2 

  Char. 385: 1 --> 0 

  Char. 390: 0 --> 1 

Seismosaurus hallorum NMMNH 3690

  Char. 239: 0 --> 1 

  Char. 353: 0 --> 1 

  Char. 354: 0 --> 1 

  Char. 414: 0 --> 1 

Barosaurus lentus YPM 429 

  Char. 135: 0 --> 1 

  Char. 138: 0 --> 1 

  Char. 141: 0 --> 1 

  Char. 255: 1 --> 0 

  Char. 264: 1 --> 0 

  Char. 277: 1 --> 0 

  Char. 280: 1 --> 0 

  Char. 300: 1 --> 0 

Barosaurus affinis YPM 419 

  No autapomorphies 

AMNH 6341 

  Char. 183: 2 --> 1 

  Char. 237: 1 --> 0 

  Char. 268: 1 --> 0 

AMNH 7530 

  No autapomorphies 

CM 11984 

  Char. 166: 0 --> 1 

  Char. 253: 1 --> 0 

  Char. 266: 1 --> 0 

  Char. 267: 1 --> 0 

AMNH 7535 

  No autapomorphies 

Cetiosauriscus stewarti NHMUK R 3078

  No autapomorphies 

Dyslocosaurus polyonychius AC 663

  Char. 441: 0 --> 1 

  Char. 455: 0 --> 1 

  Char. 467: 0 --> 1 

SMA O25-8 

  Char. 24: 0 --> 1 

  Char. 26: 0 --> 1 

  Char. 33: 0 --> 1 

  Char. 34: 0 --> 1 

  Char. 45: 0 --> 1 

  Char. 57: 0 --> 1 

  Char. 59: 0 --> 1 

  Char. 64: 0 --> 1 

  Char. 65: 0 --> 1 

  Char. 66: 0 --> 1 

  Char. 72: 0 --> 1 

  Char. 73: 0 --> 1 

  Char. 74: 0 --> 1 

  Char. 76: 0 --> 1 

  Char. 81: 0 --> 1 

  Char. 82: 0 --> 1 

  Char. 84: 0 --> 2 

  Char. 87: 0 --> 1 

  Char. 88: 0 --> 1 

  Char. 90: 0 --> 1 

  Char. 92: 0 --> 1 

  Char. 93: 0 --> 1 

  Char. 95: 0 --> 1 

  Char. 108: 0 --> 1 

SMA D16-3 

  No autapomorphies 

CM 11255 

  Char. 0: 1 --> 0 

  Char. 2: 0 --> 1 

  Char. 3: 1 --> 2 

  Char. 4: 0 --> 2 

  Char. 7: 0 --> 1 

  Char. 12: 0 --> 1 

  Char. 13: 0 --> 1 

  Char. 14: 0 --> 1 

  Char. 16: 0 --> 1 

  Char. 19: 0 --> 1 

  Char. 20: 0 --> 1 

  Char. 24: 0 --> 1 

  Char. 31: 1 --> 0 

  Char. 36: 0 --> 1 

  Char. 37: 0 --> 1 

  Char. 39: 0 --> 1 

  Char. 41: 0 --> 1 

  Char. 43: 0 --> 1 

  Char. 44: 0 --> 1 

  Char. 45: 0 --> 1 

  Char. 46: 0 --> 1 

  Char. 48: 0 --> 1 

  Char. 53: 0 --> 1 

  Char. 54: 0 --> 1 

  Char. 64: 0 --> 1 

  Char. 66: 0 --> 1 

  Char. 78: 0 --> 1 

  Char. 81: 0 --> 1 

  Char. 82: 0 --> 1 

  Char. 84: 0 --> 2 

  Char. 88: 0 --> 1 

  Char. 92: 0 --> 1 

  Char. 93: 0 --> 1 

  Char. 95: 0 --> 1 

  Char. 97: 0 --> 1 

  Char. 101: 0 --> 1 

  Char. 103: 0 --> 1 

  Char. 107: 0 --> 1 

  Char. 108: 0 --> 1 

  Char. 109: 0 --> 1 

  Char. 112: 0 --> 1 

  Char. 115: 0 --> 1 

  Char. 116: 0 --> 1 

  Char. 117: 0 --> 1 

  Char. 119: 0 --> 1 

  Char. 120: 0 --> 1 

  Char. 121: 0 --> 1 

  Char. 124: 0 --> 1 

USNM 2673 

  Char. 9: 0 --> 1 

  Char. 54: 1 --> 0 

  Char. 57: 1 --> 0 

  Char. 60: 1 --> 0 

  Char. 72: 1 --> 0 

  Char. 95: 0 --> 1 

BYU 1252-18531 

  Char. 138: 0 --> 1 

  Char. 170: 1 --> 2 

  Char. 181: 0 --> 1 

  Char. 213: 1 --> 0 

  Char. 216: 0 --> 1 

  Char. 253: 0 --> 1 

  Char. 338: 0 --> 1 

  Char. 369: 0 --> 1 

  Char. 370: 1 --> 0 

Leinkupal laticauda MMCH Pv 63-1

  Char. 313: 1 --> 0 

Node 82 

  No synapomorphies 

Node 83 

  Char. 35: 0 --> 1 

  Char. 91: 0 --> 1 

  Char. 131: 0 --> 1 

  Char. 133: 0 --> 1 

  Char. 134: 0 --> 1 

  Char. 170: 0 --> 2 

  Char. 184: 0 --> 1 

  Char. 251: 0 --> 1 

  Char. 269: 0 --> 2 

  Char. 285: 0 --> 1 

  Char. 430: 1 --> 0 

  Char. 445: 0 --> 1 

Node 84 

  Char. 21: 0 --> 1 

  Char. 108: 0 --> 1 

  Char. 214: 1 --> 0 

  Char. 286: 0 --> 1 

  Char. 397: 0 --> 1 

  Char. 419: 0 --> 1 

  Char. 452: 0 --> 1 

Node 85 

  Char. 319: 0 --> 1 

  Char. 322: 0 --> 1 

  Char. 462: 1 --> 0 

Node 86 

  Char. 448: 0 --> 1 

Node 87 (Turiasauria)

  Char. 80: 0 --> 1 

  Char. 96: 0 --> 1 

  Char. 97: 0 --> 1 

  Char. 129: 1 --> 0 

  Char. 156: 1 --> 0 

  Char. 170: 2 --> 0 

  Char. 207: 0 --> 1 

  Char. 217: 0 --> 1 

  Char. 227: 1 --> 0 

  Char. 229: 0 --> 1 

  Char. 303: 0 --> 2 

  Char. 381: 0 --> 1 

  Char. 384: 0 --> 1 

Node 88 

  Char. 408: 0 --> 1 

Node 89 

  Char. 326: 0 --> 1 

  Char. 435: 0 --> 1 

Node 90 (Camarasauridae)

  Char. 316: 0 --> 1 

Node 91 

  Char. 4: 0 --> 1 

  Char. 36: 0 --> 1 

  Char. 113: 0 --> 1 

  Char. 124: 0 --> 1 

  Char. 273: 0 --> 1 

  Char. 343: 0 --> 1 

  Char. 366: 0 --> 1 

  Char. 373: 0 --> 2 

  Char. 383: 0 --> 1 

  Char. 385: 0 --> 1 

  Char. 394: 0 --> 1 

  Char. 400: 0 --> 1 

  Char. 440: 1 --> 0 

  Char. 445: 1 --> 0 

  Char. 454: 0 --> 1 

Node 92 (Camarasaurus)

  Char. 91: 1 --> 0 

  Char. 332: 0 --> 1 

  Char. 390: 0 --> 1 

  Char. 407: 1 --> 0 

Node 93 

  Char. 364: 0 --> 1 

Node 94 

  Char. 389: 1 --> 0 

Node 95 (Dicraeosauridae)

  Char. 430: 0 --> 1 

  Char. 442: 0 --> 1 

  Char. 451: 0 --> 1 

  Char. 460: 1 --> 0 

  Char. 475: 1 --> 0 

Node 96 (Flagellicaudata)

  Char. 7: 0 --> 1 

  Char. 14: 0 --> 1 

  Char. 50: 1 --> 0 

  Char. 53: 0 --> 1 

  Char. 55: 0 --> 1 

  Char. 57: 0 --> 1 

  Char. 58: 0 --> 1 

  Char. 86: 0 --> 1 

  Char. 103: 0 --> 1 

  Char. 121: 0 --> 1 

  Char. 122: 1 --> 0 

  Char. 125: 0 --> 1 

  Char. 178: 0 --> 1 

  Char. 201: 1 --> 0 

  Char. 249: 0 --> 1 

  Char. 260: 0 --> 1 

  Char. 275: 1 --> 0 

  Char. 302: 0 --> 1 

  Char. 303: 0 --> 1 

  Char. 304: 0 --> 1 

  Char. 332: 0 --> 1 

  Char. 351: 1 --> 0 

  Char. 370: 0 --> 1 

  Char. 422: 0 --> 1 

  Char. 423: 1 --> 0 

  Char. 424: 0 --> 1 

  Char. 462: 0 --> 1 

  Char. 464: 0 --> 1 

Node 97 (Diplodocimorpha)

  Char. 281: 0 --> 1 

  Char. 299: 0 --> 1 

  Char. 316: 0 --> 1 

  Char. 317: 0 --> 1 

  Char. 408: 0 --> 1 

Node 98 (Diplodocoidea)

  Char. 213: 0 --> 1 

  Char. 214: 0 --> 1 

  Char. 427: 1 --> 0 

  Char. 453: 0 --> 1 

Node 99 (Neosauropoda)

  Char. 224: 0 --> 1 

  Char. 351: 0 --> 1 

  Char. 382: 0 --> 1 

  Char. 407: 1 --> 0 

  Char. 457: 0 --> 1 

  Char. 460: 0 --> 1 

Node 100 

  Char. 137: 1 --> 0 

  Char. 170: 2 --> 1 

  Char. 230: 0 --> 1 

  Char. 240: 0 --> 1 

  Char. 262: 0 --> 1 

Node 101 (Brachiosauridae)

  Char. 132: 0 --> 1 

  Char. 182: 0 --> 1 

  Char. 263: 0 --> 1 

  Char. 266: 01 --> 2 

  Char. 401: 1 --> 2 

  Char. 435: 0 --> 1 

Node 102 (Titanosauriformes)

  Char. 129: 1 --> 2 

  Char. 352: 0 --> 1 

  Char. 367: 1 --> 0 

  Char. 386: 1 --> 0 

  Char. 409: 0 --> 1 

  Char. 428: 0 --> 1 

  Char. 429: 1 --> 2 

Node 103 

  Char. 130: 1 --> 0 

  Char. 135: 0 --> 1 

  Char. 171: 0 --> 1 

Node 104 

  Char. 175: 0 --> 1 

  Char. 249: 1 --> 0 

  Char. 250: 0 --> 1 

  Char. 283: 0 --> 1 

Node 105 

  Char. 411: 0 --> 1 

  Char. 419: 1 --> 0 

  Char. 447: 0 --> 1 

Node 106 

  Char. 306: 0 --> 1 

  Char. 331: 0 --> 1 

Node 107 (Diplodocinae)

  Char. 266: 0 --> 1 

  Char. 272: 0 --> 1 

  Char. 299: 1 --> 3 

  Char. 420: 0 --> 1 

  Char. 441: 0 --> 1 

  Char. 458: 0 --> 1 

Node 108 (Diplodocidae)

  Char. 22: 0 --> 1 

  Char. 24: 0 --> 1 

  Char. 49: 0 --> 1 

  Char. 126: 0 --> 1 

  Char. 198: 0 --> 1 

  Char. 207: 0 --> 1 

  Char. 223: 1 --> 2 

  Char. 257: 0 --> 1 

  Char. 258: 0 --> 1 

  Char. 262: 1 --> 0 

  Char. 313: 0 --> 1 

  Char. 315: 0 --> 1 

  Char. 318: 0 --> 1 

  Char. 320: 0 --> 1 

  Char. 382: 1 --> 0 

  Char. 391: 0 --> 1 

  Char. 427: 0 --> 1 

Node 109 (Somphospondyli)

  Char. 184: 1 --> 0 

  Char. 185: 0 --> 1 

  Char. 188: 1 --> 0 

  Char. 227: 1 --> 2 

  Char. 232: 0 --> 1 

  Char. 262: 1 --> 0 

  Char. 273: 1 --> 0 

  Char. 365: 0 --> 1 

  Char. 366: 1 --> 0 

  Char. 370: 0 --> 1 

Node 110 (Limaysaurinae)

  Char. 184: 1 --> 0 

  Char. 318: 0 --> 1 

  Char. 363: 0 --> 1 

Node 111 

  Char. 335: 0 --> 1 

  Char. 416: 0 --> 1 

  Char. 418: 0 --> 1 

  Char. 420: 0 --> 1 

Node 112 (Rebbachisauridae)

  Char. 188: 1 --> 0 

  Char. 196: 0 --> 1 

  Char. 324: 0 --> 1 

  Char. 333: 0 --> 2 

  Char. 417: 0 --> 1 

  Char. 459: 0 --> 1 

  Char. 461: 0 --> 1 

Node 113 (Nigersaurinae)

  Char. 261: 0 --> 1 

  Char. 291: 0 --> 1 

  Char. 434: 0 --> 1 

Node 114 

  Char. 279: 1 --> 0 

  Char. 429: 1 --> 0 

Node 115 (Brontosaurus)

  Char. 162: 0 --> 1 

  Char. 183: 1 --> 0 

  Char. 269: 1 --> 0 

  Char. 287: 0 --> 1 

  Char. 292: 0 --> 1 

  Char. 349: 0 --> 1 

  Char. 450: 0 --> 1 

Node 116 

  Char. 222: 0 --> 1 

  Char. 258: 1 --> 0 

Node 117 

  Char. 137: 0 --> 1 

  Char. 158: 0 --> 1 

  Char. 173: 0 --> 1 

  Char. 178: 1 --> 0 

  Char. 224: 1 --> 0 

  Char. 237: 0 --> 1 

  Char. 249: 1 --> 0 

  Char. 253: 1 --> 0 

  Char. 295: 0 --> 1 

Node 118 (Apatosaurinae)

  Char. 134: 0 --> 1 

  Char. 185: 0 --> 1 

  Char. 200: 0 --> 1 

  Char. 215: 0 --> 1 

  Char. 219: 0 --> 1 

  Char. 279: 0 --> 1 

  Char. 353: 0 --> 1 

  Char. 371: 0 --> 1 

Node 119 

  Char. 133: 1 --> 0 

  Char. 167: 1 --> 2 

  Char. 178: 1 --> 0 

  Char. 254: 0 --> 1 

  Char. 267: 1 --> 0 

  Char. 280: 0 --> 1 

Node 120 

  Char. 129: 1 --> 0 

  Char. 131: 1 --> 0 

  Char. 169: 0 --> 1 

  Char. 176: 0 --> 1 

  Char. 224: 1 --> 0 

  Char. 226: 1 --> 0 

  Char. 227: 1 --> 0 

  Char. 245: 0 --> 1 

  Char. 251: 1 --> 0 

Node 121 

  Char. 205: 0 --> 1 

  Char. 252: 0 --> 1 

  Char. 259: 0 --> 1 

  Char. 269: 0 --> 1 

  Char. 292: 1 --> 0 

  Char. 327: 1 --> 0 

  Char. 328: 1 --> 0 

  Char. 360: 1 --> 0 

  Char. 364: 0 --> 1 

  Char. 368: 0 --> 1 

Node 122 

  Char. 162: 1 --> 0 

  Char. 258: 0 --> 1 

  Char. 263: 0 --> 1 

  Char. 389: 0 --> 1 

Node 123 

  Char. 273: 1 --> 0 

  Char. 387: 0 --> 1 

Node 124 

  Char. 270: 0 --> 1 

  Char. 272: 0 --> 1 

Node 125 

  Char. 266: 0 --> 1 

  Char. 437: 0 --> 1 

Node 126 (Apatosaurus louisae)

  Char. 221: 0 --> 1 

Node 127 (Apatosaurus)

  Char. 207: 1 --> 0 

  Char. 217: 0 --> 1 

Node 128 (Brontosaurus excelsus)

  Char. 309: 0 --> 1 

Node 129 

  Char. 127: 1 --> 2 

  Char. 167: 1 --> 0 

  Char. 187: 0 --> 1 

  Char. 236: 0 --> 1 

  Char. 425: 0 --> 1 

Node 130 (Galeamopus hayi)

  Char. 165: 1 --> 0 

Node 131 

  Char. 34: 0 --> 1 

  Char. 59: 1 --> 2 

  Char. 66: 0 --> 1 

  Char. 78: 1 --> 0 

  Char. 89: 0 --> 1 

Node 132 

  Char. 11: 0 --> 1 

Node 133 (Galeamopus)

  Char. 61: 1 --> 0 

  Char. 145: 0 --> 1 

  Char. 147: 0 --> 1 

  Char. 150: 0 --> 1 

Node 134 

  Char. 297: 0 --> 1 

Node 135 

  Char. 314: 0 --> 1 

Node 136 

  Char. 323: 0 --> 1 

  Char. 443: 1 --> 0 

  Char. 444: 0 --> 1 

  Char. 454: 0 --> 1 

Node 137 (Supersaurus vivianae)

  Char. 183: 1 --> 2 

Node 138 (Kaatedocus)

  Char. 130: 1 --> 0 

  Char. 177: 0 --> 1 

  Char. 182: 0 --> 1 

  Char. 201: 0 --> 1 

  Char. 202: 1 --> 0 

  Char. 204: 1 --> 0 

  Char. 210: 0 --> 1 

  Char. 211: 0 --> 1 

Node 139 

  Char. 156: 1 --> 0 

  Char. 163: 0 --> 1 

Node 140 

  Char. 133: 1 --> 0 

  Char. 139: 1 --> 2 

  Char. 183: 1 --> 0 

Node 141 

  Char. 153: 1 --> 0 

  Char. 195: 0 --> 1 

  Char. 237: 0 --> 1 

  Char. 268: 0 --> 1 

  Char. 280: 0 --> 1 

  Char. 332: 1 --> 0 

  Char. 334: 0 --> 1 

  Char. 366: 1 --> 0 

  Char. 380: 0 --> 1 

  Char. 439: 0 --> 1 

  Char. 460: 1 --> 0 

Node 142 (Tornieria)

  Char. 378: 0 --> 1 

  Char. 417: 0 --> 1 

  Char. 425: 0 --> 1 

Node 143 (Diplodocus carnegii)

  Char. 138: 0 --> 1 

  Char. 198: 1 --> 0 

  Char. 246: 0 --> 1 

  Char. 276: 0 --> 1 

  Char. 294: 1 --> 0 

  Char. 420: 1 --> 0 

Node 144 (Diplodocus)

  Char. 181: 1 --> 0 

  Char. 264: 1 --> 0 

  Char. 279: 0 --> 1 

  Char. 299: 3 --> 4 

  Char. 307: 0 --> 1 

  Char. 330: 0 --> 1 

  Char. 335: 0 --> 1 

  Char. 467: 0 --> 1 

Node 145 (Diplodocus hallorum)

  Char. 233: 0 --> 1 

  Char. 336: 0 --> 1 

  Char. 339: 0 --> 1 

  Char. 342: 0 --> 1 

  Char. 356: 0 --> 1 

Node 146 

  Char. 257: 2 --> 1 

Node 147 

  Char. 425: 0 --> 1 

Node 148 

  Char. 136: 0 --> 1 

  Char. 182: 0 --> 1 

  Char. 187: 0 --> 1 

  Char. 199: 0 --> 1 

Node 149 

  Char. 171: 0 --> 1 

  Char. 183: 0 --> 2 

Node 150 (Barosaurus)

  Char. 165: 1 --> 2 

Node 151 

  Char. 457: 0 --> 1 

  Char. 464: 0 --> 1 

  Char. 470: 0 --> 1 

Node 152 

  No synapomorphies 

 

### P_iw

Tree 60002 

Shunosaurus lii 

  No autapomorphies 

Spinophorosaurus nigerensis 

  Char. 0: 1 --> 2 

  Char. 25: 0 --> 1 

  Char. 28: 0 --> 1 

  Char. 63: 0 --> 1 

  Char. 65: 0 --> 1 

  Char. 90: 0 --> 1 

  Char. 97: 0 --> 1 

  Char. 160: 0 --> 1 

  Char. 165: 0 --> 1 

  Char. 179: 0 --> 1 

  Char. 180: 0 --> 1 

  Char. 181: 0 --> 1 

  Char. 186: 0 --> 1 

  Char. 189: 0 --> 1 

  Char. 195: 0 --> 1 

  Char. 199: 0 --> 1 

  Char. 202: 0 --> 1 

  Char. 273: 0 --> 1 

  Char. 298: 1 --> 0 

  Char. 326: 0 --> 1 

  Char. 331: 0 --> 1 

  Char. 333: 0 --> 2 

  Char. 366: 0 --> 1 

  Char. 379: 1 --> 0 

Omeisaurus 

  Char. 72: 0 --> 1 

  Char. 76: 0 --> 1 

  Char. 165: 0 --> 2 

  Char. 175: 0 --> 1 

  Char. 197: 0 --> 1 

  Char. 278: 1 --> 0 

  Char. 284: 0 --> 1 

  Char. 327: 0 --> 1 

  Char. 342: 0 --> 1 

  Char. 358: 0 --> 1 

  Char. 372: 0 --> 1 

  Char. 386: 1 --> 2 

  Char. 392: 0 --> 1 

  Char. 413: 0 --> 1 

  Char. 475: 1 --> 0 

Mamenchisaurus 

  Char. 57: 0 --> 1 

  Char. 62: 1 --> 0 

  Char. 73: 0 --> 1 

  Char. 125: 0 --> 1 

  Char. 129: 01 --> 2 

  Char. 170: 2 --> 1 

  Char. 206: 0 --> 1 

  Char. 227: 01 --> 2 

  Char. 253: 0 --> 1 

  Char. 272: 0 --> 1 

  Char. 294: 0 --> 1 

  Char. 303: 0 --> 3 

  Char. 357: 1 --> 0 

  Char. 369: 0 --> 1 

  Char. 398: 0 --> 1 

  Char. 425: 0 --> 1 

  Char. 465: 1 --> 0 

  Char. 474: 0 --> 1 

Jobaria tiguidensis 

  Char. 122: 1 --> 0 

  Char. 134: 1 --> 0 

  Char. 140: 0 --> 1 

  Char. 152: 0 --> 2 

  Char. 156: 1 --> 0 

  Char. 159: 0 --> 1 

  Char. 180: 0 --> 1 

  Char. 183: 0 --> 1 

  Char. 184: 1 --> 0 

  Char. 189: 0 --> 1 

  Char. 197: 0 --> 1 

  Char. 212: 0 --> 1 

  Char. 223: 1 --> 0 

  Char. 251: 1 --> 0 

  Char. 265: 1 --> 0 

  Char. 269: 2 --> 0 

  Char. 275: 1 --> 0 

  Char. 278: 1 --> 0 

  Char. 352: 0 --> 1 

  Char. 392: 0 --> 1 

  Char. 407: 1 --> 0 

  Char. 432: 0 --> 1 

  Char. 435: 0 --> 1 

  Char. 444: 0 --> 1 

Turiasaurus riodevensis 

  No autapomorphies 

Losillasaurus giganteus type 

  Char. 260: 0 --> 1 

  Char. 261: 0 --> 1 

  Char. 309: 0 --> 1 

  Char. 386: 1 --> 0 

Camarasaurus 

  Char. 89: 0 --> 1 

  Char. 260: 0 --> 1 

  Char. 351: 0 --> 1 

  Char. 386: 1 --> 2 

  Char. 432: 0 --> 1 

  Char. 437: 0 --> 1 

Amphicoelias latus AMNH 5765 

  No autapomorphies 

Apatosaurus grandis YPM 1901 

  Char. 340: 0 --> 1 

  Char. 344: 0 --> 1 

  Char. 348: 0 --> 1 

  Char. 352: 0 --> 1 

  Char. 361: 2 --> 1 

  Char. 363: 0 --> 1 

  Char. 373: 2 --> 1 

  Char. 429: 1 --> 2 

  Char. 450: 0 --> 1 

  Char. 458: 0 --> 1 

  Char. 465: 1 --> 0 

Dystrophaeus viaemalae USNM 2364

  Char. 369: 0 --> 1 

Lourinhasaurus alenquerensis lectotype

  Char. 303: 0 --> 1 

  Char. 305: 0 --> 1 

  Char. 369: 0 --> 1 

  Char. 379: 1 --> 0 

  Char. 392: 0 --> 1 

  Char. 409: 0 --> 1 

  Char. 420: 0 --> 1 

  Char. 423: 1 --> 0 

  Char. 425: 0 --> 1 

Giraffatitan brancai 

  Char. 62: 1 --> 0 

  Char. 82: 1 --> 0 

  Char. 117: 0 --> 1 

  Char. 165: 0 --> 2 

  Char. 233: 0 --> 1 

  Char. 260: 0 --> 1 

  Char. 261: 0 --> 1 

  Char. 303: 0 --> 1 

  Char. 305: 0 --> 1 

  Char. 312: 0 --> 1 

  Char. 372: 0 --> 1 

  Char. 382: 1 --> 0 

  Char. 385: 1 --> 0 

SMA 0009 

  Char. 132: 0 --> 1 

  Char. 157: 0 --> 1 

  Char. 167: 1 --> 0 

  Char. 172: 0 --> 1 

  Char. 199: 0 --> 1 

  Char. 217: 0 --> 1 

  Char. 238: 0 --> 1 

  Char. 254: 0 --> 1 

  Char. 261: 0 --> 1 

  Char. 265: 1 --> 0 

  Char. 278: 1 --> 0 

  Char. 284: 0 --> 2 

  Char. 294: 0 --> 2 

  Char. 303: 0 --> 1 

  Char. 320: 0 --> 1 

  Char. 329: 0 --> 1 

  Char. 333: 0 --> 1 

  Char. 341: 0 --> 1 

  Char. 363: 0 --> 1 

  Char. 371: 0 --> 1 

  Char. 393: 1 --> 0 

  Char. 395: 0 --> 1 

  Char. 412: 0 --> 1 

  Char. 431: 1 --> 0 

  Char. 433: 0 --> 1 

  Char. 439: 0 --> 1 

  Char. 465: 1 --> 0 

  Char. 475: 1 --> 0 

Brachiosaurus altithorax 

  Char. 25: 0 --> 1 

  Char. 30: 0 --> 1 

  Char. 326: 0 --> 1 

  Char. 404: 0 --> 1 

  Char. 412: 0 --> 1 

  Char. 431: 1 --> 0 

  Char. 433: 0 --> 1 

Australodocus bohetii type 

  Char. 129: 1 --> 2 

  Char. 166: 0 --> 1 

  Char. 170: 2 --> 1 

  Char. 173: 0 --> 1 

  Char. 178: 1 --> 0 

  Char. 188: 1 --> 0 

  Char. 217: 1 --> 0 

Ligabuesaurus leanzai 

  Char. 127: 1 --> 2 

  Char. 140: 0 --> 1 

  Char. 197: 0 --> 1 

  Char. 242: 0 --> 1 

  Char. 363: 0 --> 1 

  Char. 369: 0 --> 1 

Isisaurus colberti 

  Char. 191: 1 --> 0 

  Char. 235: 0 --> 1 

  Char. 358: 0 --> 1 

  Char. 417: 0 --> 1 

Haplocanthosaurus priscus 

  Char. 158: 0 --> 1 

  Char. 166: 0 --> 1 

  Char. 173: 0 --> 1 

  Char. 176: 0 --> 1 

  Char. 184: 1 --> 0 

  Char. 186: 0 --> 1 

  Char. 200: 0 --> 1 

  Char. 223: 1 --> 0 

  Char. 227: 1 --> 0 

  Char. 236: 0 --> 1 

  Char. 241: 0 --> 1 

  Char. 246: 0 --> 1 

  Char. 263: 0 --> 1 

  Char. 287: 0 --> 1 

  Char. 296: 0 --> 1 

  Char. 323: 0 --> 1 

  Char. 348: 0 --> 1 

  Char. 372: 0 --> 1 

  Char. 411: 0 --> 1 

  Char. 420: 0 --> 1 

Limaysaurus tessonei 

  Char. 186: 0 --> 1 

  Char. 296: 0 --> 1 

  Char. 305: 0 --> 1 

  Char. 349: 0 --> 1 

Cathartesaura anaerobica 

  Char. 198: 0 --> 1 

  Char. 320: 0 --> 1 

  Char. 338: 1 --> 0 

  Char. 342: 0 --> 1 

Zapalasaurus bonapartei 

  Char. 308: 0 --> 1 

  Char. 390: 0 --> 1 

Nigersaurus taqueti 

  Char. 106: 0 --> 1 

  Char. 111: 0 --> 1 

  Char. 113: 1 --> 0 

  Char. 165: 0 --> 1 

  Char. 170: 1 --> 2 

  Char. 182: 0 --> 1 

  Char. 184: 1 --> 2 

  Char. 213: 1 --> 0 

  Char. 237: 0 --> 1 

  Char. 257: 0 --> 1 

  Char. 327: 0 --> 1 

Demandasaurus darwini 

  Char. 113: 1 --> 2 

  Char. 118: 1 --> 0 

  Char. 193: 0 --> 1 

  Char. 272: 0 --> 1 

  Char. 281: 1 --> 2 

  Char. 324: 1 --> 0 

  Char. 326: 0 --> 1 

  Char. 329: 0 --> 1 

  Char. 332: 0 --> 1 

  Char. 333: 2 --> 3 

  Char. 429: 1 --> 2 

Amphicoelias altus AMNH 5764 

  Char. 255: 0 --> 1 

  Char. 264: 0 --> 1 

  Char. 267: 1 --> 0 

  Char. 274: 1 --> 0 

  Char. 426: 12 --> 0 

Dicraeosaurus hansemanni 

  Char. 32: 0 --> 1 

  Char. 46: 0 --> 1 

  Char. 59: 1 --> 0 

  Char. 132: 0 --> 1 

  Char. 137: 0 --> 1 

  Char. 170: 1 --> 0 

  Char. 181: 1 --> 0 

  Char. 211: 0 --> 1 

  Char. 216: 0 --> 1 

  Char. 429: 1 --> 0 

  Char. 443: 1 --> 0 

Brachytrachelopan mesai 

  Char. 197: 0 --> 1 

  Char. 213: 1 --> 0 

  Char. 407: 0 --> 1 

Amargasaurus cazaui 

  Char. 56: 0 --> 1 

  Char. 62: 1 --> 0 

  Char. 80: 0 --> 1 

  Char. 96: 0 --> 1 

  Char. 183: 0 --> 1 

  Char. 186: 0 --> 1 

  Char. 188: 1 --> 0 

  Char. 206: 1 --> 0 

  Char. 209: 1 --> 0 

  Char. 242: 0 --> 1 

  Char. 243: 1 --> 0 

  Char. 257: 0 --> 1 

  Char. 303: 1 --> 0 

  Char. 381: 0 --> 1 

  Char. 386: 1 --> 0 

  Char. 387: 1 --> 0 

  Char. 390: 0 --> 1 

  Char. 411: 0 --> 1 

  Char. 431: 1 --> 0 

  Char. 432: 0 --> 1 

Suuwassea emilieae ANS 21122 

  No autapomorphies 

Apatosaurus ajax YPM 1860 

  Char. 132: 1 --> 0 

  Char. 163: 0 --> 1 

  Char. 167: 1 --> 0 

  Char. 168: 0 --> 1 

  Char. 186: 0 --> 1 

  Char. 196: 1 --> 0 

  Char. 197: 0 --> 1 

  Char. 207: 1 --> 0 

  Char. 237: 0 --> 1 

  Char. 257: 1 --> 0 

  Char. 270: 1 --> 0 

  Char. 278: 0 --> 1 

  Char. 291: 0 --> 1 

  Char. 367: 1 --> 0 

Apatosaurus laticollis YPM 1861 

  Char. 127: 1 --> 2 

  Char. 176: 0 --> 1 

  Char. 197: 0 --> 1 

  Char. 200: 1 --> 0 

Brontosaurus excelsus YPM 1980 

  Char. 237: 1 --> 0 

  Char. 354: 1 --> 0 

Brontosaurus amplus YPM 1981 

  Char. 260: 1 --> 0 

  Char. 353: 1 --> 0 

  Char. 373: 1 --> 0 

  Char. 374: 1 --> 0 

  Char. 375: 0 --> 1 

  Char. 411: 0 --> 1 

  Char. 421: 1 --> 0 

  Char. 435: 0 --> 1 

Apatosaurus louisae type 

  Char. 187: 0 --> 1 

  Char. 310: 0 --> 1 

Atlantosaurus immanis YPM 1840 

  Char. 141: 0 --> 1 

  Char. 217: 0 --> 1 

Elosaurus parvus CM 566 

  Char. 134: 1 --> 0 

  Char. 137: 1 --> 0 

  Char. 226: 1 --> 0 

  Char. 251: 1 --> 0 

  Char. 253: 0 --> 1 

  Char. 257: 1 --> 0 

  Char. 260: 1 --> 0 

  Char. 365: 1 --> 0 

  Char. 385: 1 --> 0 

  Char. 436: 0 --> 1 

  Char. 447: 0 --> 1 

UW 15556 

  Char. 182: 0 --> 1 

  Char. 201: 0 --> 1 

  Char. 208: 1 --> 0 

  Char. 241: 0 --> 1 

  Char. 304: 1 --> 0 

  Char. 388: 1 --> 0 

Apatosaurus minimus AMNH 675 

  Char. 287: 0 --> 1 

  Char. 423: 1 --> 0 

  Char. 424: 0 --> 1 

Eobrontosaurus yahnahpin Tate-001

  Char. 127: 1 --> 2 

  Char. 158: 1 --> 0 

  Char. 217: 0 --> 1 

  Char. 244: 1 --> 0 

  Char. 264: 0 --> 1 

  Char. 320: 1 --> 0 

  Char. 338: 0 --> 1 

  Char. 371: 1 --> 0 

  Char. 392: 0 --> 1 

  Char. 393: 1 --> 0 

  Char. 398: 0 --> 1 

NSMT-PV 20375 

  Char. 182: 0 --> 1 

  Char. 242: 0 --> 1 

  Char. 417: 0 --> 1 

  Char. 419: 1 --> 0 

  Char. 430: 0 --> 1 

  Char. 435: 0 --> 1 

LACM 52844 

  Char. 133: 1 --> 0 

  Char. 157: 0 --> 1 

  Char. 187: 0 --> 1 

  Char. 295: 1 --> 0 

  Char. 303: 1 --> 0 

  Char. 331: 0 --> 1 

  Char. 381: 0 --> 1 

  Char. 432: 0 --> 1 

AMNH 460 

  Char. 141: 0 --> 1 

  Char. 267: 1 --> 0 

  Char. 270: 0 --> 1 

  Char. 407: 0 --> 1 

  Char. 411: 0 --> 1 

CM 3378 

  Char. 171: 1 --> 0 

  Char. 329: 0 --> 1 

  Char. 338: 0 --> 1 

FMNH P25112 

  Char. 225: 1 --> 0 

  Char. 258: 0 --> 1 

  Char. 269: 0 --> 1 

  Char. 295: 1 --> 0 

  Char. 308: 0 --> 1 

  Char. 323: 0 --> 1 

  Char. 411: 0 --> 1 

  Char. 415: 1 --> 0 

  Char. 419: 1 --> 0 

  Char. 429: 0 --> 2 

  Char. 430: 0 --> 1 

ML 418 

  Char. 178: 1 --> 0 

SMA 0087 

  No autapomorphies 

Supersaurus vivianae BYU  

  Char. 258: 1 --> 0 

  Char. 274: 1 --> 0 

WDC DMJ-021 

  Char. 165: 1 --> 2 

  Char. 172: 0 --> 1 

  Char. 174: 0 --> 1 

  Char. 257: 1 --> 2 

Dinheirosaurus lourinhanensis ML 414

  Char. 125: 1 --> 0 

  Char. 138: 0 --> 1 

  Char. 187: 0 --> 1 

  Char. 229: 0 --> 1 

  Char. 280: 0 --> 1 

  Char. 304: 1 --> 0 

Kaatedocus siberi SMA 0004 

  Char. 71: 0 --> 1 

  Char. 85: 0 --> 1 

  Char. 166: 0 --> 1 

Tornieria africana holotype 

  Char. 330: 0 --> 1 

Tornieria africana skeleton k 

  No autapomorphies 

Diplodocus longus YPM 1920 

  Char. 305: 0 --> 1 

  Char. 329: 0 --> 1 

  Char. 331: 0 --> 1 

  Char. 333: 0 --> 2 

  Char. 334: 0 --> 1 

  Char. 338: 0 --> 1 

  Char. 343: 0 --> 1 

Diplodocus lacustris YPM 1922 

  Char. 116: 0 --> 1 

  Char. 118: 0 --> 1 

  Char. 119: 0 --> 1 

  Char. 120: 0 --> 1 

  Char. 124: 0 --> 1 

Diplodocus carnegii CM 84 

  Char. 407: 1 --> 0 

  Char. 411: 1 --> 0 

Diplodocus carnegii CM 94 

  Char. 134: 0 --> 1 

  Char. 186: 0 --> 1 

  Char. 200: 0 --> 1 

  Char. 237: 1 --> 0 

  Char. 240: 1 --> 0 

  Char. 353: 0 --> 1 

  Char. 365: 0 --> 1 

  Char. 366: 0 --> 1 

AMNH 223 

  Char. 277: 1 --> 0 

  Char. 337: 0 --> 1 

  Char. 358: 0 --> 1 

  Char. 368: 0 --> 1 

  Char. 411: 1 --> 0 

USNM 10865 

  Char. 340: 0 --> 1 

DMNS 1494 

  Char. 420: 1 --> 0 

  Char. 421: 0 --> 1 

  Char. 447: 1 --> 0 

AMNH 969 

  Char. 82: 1 --> 0 

  Char. 111: 0 --> 1 

  Char. 149: 0 --> 1 

CM 11161 

  Char. 0: 1 --> 0 

  Char. 1: 0 --> 1 

  Char. 2: 0 --> 1 

  Char. 3: 1 --> 2 

  Char. 4: 0 --> 2 

  Char. 7: 0 --> 1 

  Char. 12: 0 --> 1 

  Char. 13: 0 --> 1 

  Char. 14: 0 --> 1 

  Char. 16: 0 --> 1 

  Char. 17: 0 --> 1 

  Char. 19: 0 --> 1 

  Char. 20: 0 --> 1 

  Char. 22: 0 --> 1 

  Char. 23: 0 --> 1 

  Char. 24: 0 --> 1 

  Char. 26: 0 --> 1 

  Char. 30: 0 --> 1 

  Char. 31: 1 --> 0 

  Char. 33: 0 --> 1 

  Char. 34: 0 --> 1 

  Char. 36: 0 --> 1 

  Char. 37: 0 --> 1 

  Char. 39: 0 --> 1 

  Char. 40: 0 --> 1 

  Char. 43: 0 --> 1 

  Char. 44: 0 --> 1 

  Char. 45: 0 --> 1 

  Char. 48: 0 --> 1 

  Char. 52: 0 --> 1 

  Char. 53: 0 --> 1 

  Char. 54: 0 --> 1 

  Char. 55: 0 --> 1 

  Char. 59: 0 --> 1 

  Char. 62: 0 --> 1 

  Char. 67: 0 --> 1 

  Char. 72: 0 --> 1 

  Char. 76: 0 --> 1 

  Char. 78: 0 --> 1 

  Char. 81: 0 --> 1 

  Char. 82: 0 --> 1 

  Char. 84: 0 --> 2 

  Char. 86: 0 --> 1 

  Char. 88: 0 --> 1 

  Char. 90: 0 --> 1 

  Char. 91: 0 --> 1 

  Char. 92: 0 --> 1 

  Char. 93: 0 --> 1 

  Char. 98: 0 --> 1 

  Char. 101: 0 --> 1 

  Char. 102: 0 --> 1 

  Char. 103: 0 --> 1 

  Char. 107: 0 --> 1 

  Char. 108: 0 --> 1 

  Char. 109: 0 --> 1 

  Char. 110: 0 --> 1 

  Char. 112: 0 --> 2 

  Char. 113: 0 --> 1 

  Char. 115: 0 --> 1 

  Char. 116: 0 --> 1 

  Char. 118: 0 --> 1 

  Char. 119: 0 --> 1 

  Char. 120: 0 --> 1 

  Char. 121: 0 --> 1 

  Char. 124: 0 --> 1 

CM 3452 

  Char. 79: 1 --> 0 

  Char. 88: 1 --> 0 

  Char. 134: 0 --> 1 

  Char. 173: 0 --> 1 

USNM 2672 

  Char. 0: 1 --> 0 

  Char. 1: 0 --> 1 

  Char. 2: 0 --> 1 

  Char. 3: 1 --> 2 

  Char. 4: 0 --> 2 

  Char. 7: 0 --> 1 

  Char. 12: 0 --> 1 

  Char. 13: 0 --> 1 

  Char. 14: 0 --> 1 

  Char. 16: 0 --> 1 

  Char. 17: 0 --> 1 

  Char. 19: 0 --> 1 

  Char. 20: 0 --> 1 

  Char. 22: 0 --> 1 

  Char. 23: 0 --> 1 

  Char. 24: 0 --> 1 

  Char. 26: 0 --> 1 

  Char. 30: 0 --> 1 

  Char. 31: 1 --> 0 

  Char. 33: 0 --> 1 

  Char. 35: 0 --> 1 

  Char. 36: 0 --> 1 

  Char. 37: 0 --> 1 

  Char. 39: 0 --> 1 

  Char. 40: 0 --> 1 

  Char. 41: 0 --> 1 

  Char. 43: 0 --> 1 

  Char. 44: 0 --> 1 

  Char. 45: 0 --> 1 

  Char. 48: 0 --> 1 

  Char. 52: 0 --> 1 

  Char. 53: 0 --> 1 

  Char. 54: 0 --> 1 

  Char. 55: 0 --> 1 

  Char. 59: 0 --> 1 

  Char. 62: 0 --> 1 

  Char. 67: 0 --> 1 

  Char. 72: 0 --> 1 

  Char. 74: 0 --> 1 

  Char. 81: 0 --> 1 

  Char. 84: 0 --> 2 

  Char. 86: 0 --> 1 

  Char. 87: 0 --> 1 

  Char. 88: 0 --> 1 

  Char. 91: 0 --> 1 

  Char. 92: 0 --> 1 

  Char. 93: 0 --> 1 

  Char. 99: 1 --> 0 

  Char. 101: 0 --> 1 

  Char. 103: 0 --> 1 

  Char. 107: 0 --> 1 

  Char. 108: 0 --> 1 

  Char. 109: 0 --> 1 

  Char. 110: 0 --> 1 

  Char. 112: 0 --> 2 

  Char. 113: 0 --> 1 

  Char. 115: 0 --> 1 

  Char. 116: 0 --> 1 

  Char. 118: 0 --> 1 

  Char. 119: 0 --> 1 

  Char. 120: 0 --> 1 

  Char. 121: 0 --> 1 

  Char. 124: 0 --> 1 

  Char. 143: 0 --> 1 

  Char. 144: 0 --> 1 

WDC FS001A 

  Char. 356: 0 --> 1 

  Char. 450: 0 --> 1 

  Char. 467: 0 --> 1 

Galeamopus hayi HMNS 175 

  Char. 133: 1 --> 0 

  Char. 138: 0 --> 1 

Galeamopus shellensis SMA 0011 

  Char. 82: 1 --> 0 

  Char. 87: 1 --> 0 

  Char. 139: 1 --> 2 

  Char. 153: 1 --> 2 

  Char. 161: 0 --> 1 

  Char. 181: 1 --> 0 

  Char. 185: 0 --> 1 

  Char. 190: 0 --> 1 

  Char. 225: 1 --> 0 

  Char. 246: 0 --> 1 

  Char. 278: 1 --> 0 

  Char. 282: 1 --> 0 

  Char. 379: 1 --> 2 

  Char. 385: 1 --> 0 

  Char. 390: 0 --> 1 

Seismosaurus hallorum NMMNH 369 

  Char. 239: 0 --> 1 

  Char. 353: 0 --> 1 

  Char. 354: 0 --> 1 

  Char. 414: 0 --> 1 

Barosaurus lentus YPM 429 

  Char. 135: 0 --> 1 

  Char. 136: 0 --> 1 

  Char. 138: 0 --> 1 

  Char. 141: 0 --> 1 

  Char. 174: 0 --> 1 

  Char. 183: 0 --> 2 

  Char. 187: 0 --> 1 

  Char. 199: 0 --> 1 

  Char. 200: 0 --> 1 

  Char. 255: 1 --> 0 

  Char. 264: 1 --> 0 

  Char. 277: 1 --> 0 

  Char. 280: 1 --> 0 

  Char. 300: 1 --> 0 

Barosaurus affinis YPM 419 

  No autapomorphies 

AMNH 6341 

  Char. 136: 0 --> 1 

  Char. 183: 0 --> 1 

  Char. 187: 0 --> 1 

  Char. 199: 0 --> 1 

  Char. 237: 1 --> 0 

  Char. 268: 1 --> 0 

AMNH 7530 

  No autapomorphies 

CM 11984 

  Char. 166: 0 --> 1 

  Char. 183: 0 --> 2 

  Char. 253: 1 --> 0 

  Char. 257: 2 --> 1 

  Char. 266: 1 --> 0 

  Char. 267: 1 --> 0 

AMNH 7535 

  Char. 49: 1 --> 0 

  Char. 157: 0 --> 1 

Cetiosauriscus stewarti NHMUK R3078

  No autapomorphies 

Dyslocosaurus polyonychius AC 663

  Char. 441: 0 --> 1 

  Char. 455: 0 --> 1 

  Char. 467: 0 --> 1 

SMA O25-8 

  Char. 77: 0 --> 1 

  Char. 87: 0 --> 1 

  Char. 99: 0 --> 1 

SMA D16-3 

  No autapomorphies 

CM 11255 

  Char. 17: 1 --> 0 

  Char. 51: 1 --> 0 

  Char. 76: 1 --> 0 

  Char. 89: 0 --> 1 

  Char. 90: 1 --> 0 

  Char. 110: 1 --> 0 

  Char. 117: 0 --> 1 

USNM 2673 

  Char. 9: 0 --> 1 

  Char. 16: 1 --> 0 

  Char. 54: 1 --> 0 

  Char. 57: 1 --> 0 

  Char. 60: 1 --> 0 

  Char. 72: 1 --> 0 

  Char. 95: 0 --> 1 

BYU 1252-18531 

  Char. 138: 0 --> 1 

  Char. 170: 1 --> 2 

  Char. 181: 0 --> 1 

  Char. 213: 1 --> 0 

  Char. 216: 0 --> 1 

  Char. 253: 0 --> 1 

  Char. 338: 0 --> 1 

  Char. 369: 0 --> 1 

  Char. 370: 1 --> 0 

Leinkupal laticauda MMCH Pv 63-1  

  Char. 313: 1 --> 0 

Node 82 

  No synapomorphies 

Node 83 

  Char. 35: 0 --> 1 

  Char. 91: 0 --> 1 

  Char. 131: 0 --> 1 

  Char. 133: 0 --> 1 

  Char. 134: 0 --> 1 

  Char. 170: 0 --> 2 

  Char. 184: 0 --> 1 

  Char. 251: 0 --> 1 

  Char. 269: 0 --> 2 

  Char. 285: 0 --> 1 

  Char. 430: 1 --> 0 

  Char. 445: 0 --> 1 

Node 84 

  Char. 21: 0 --> 1 

  Char. 108: 0 --> 1 

  Char. 214: 1 --> 0 

  Char. 286: 0 --> 1 

  Char. 397: 0 --> 1 

  Char. 419: 0 --> 1 

  Char. 452: 0 --> 1 

Node 85 

  Char. 319: 0 --> 1 

  Char. 322: 0 --> 1 

  Char. 462: 1 --> 0 

Node 86 

  Char. 448: 0 --> 1 

Node 87 (Turiasauria)

  Char. 80: 0 --> 1 

  Char. 96: 0 --> 1 

  Char. 97: 0 --> 1 

  Char. 129: 1 --> 0 

  Char. 156: 1 --> 0 

  Char. 170: 2 --> 0 

  Char. 207: 0 --> 1 

  Char. 217: 0 --> 1 

  Char. 227: 1 --> 0 

  Char. 229: 0 --> 1 

  Char. 303: 0 --> 2 

  Char. 381: 0 --> 1 

  Char. 384: 0 --> 1 

Node 88 

  Char. 408: 0 --> 1 

Node 89 

  Char. 326: 0 --> 1 

  Char. 435: 0 --> 1 

Node 90 (Camarasauridae)

  Char. 316: 0 --> 1 

Node 91 

  Char. 4: 0 --> 1 

  Char. 36: 0 --> 1 

  Char. 113: 0 --> 1 

  Char. 124: 0 --> 1 

  Char. 273: 0 --> 1 

  Char. 343: 0 --> 1 

  Char. 366: 0 --> 1 

  Char. 373: 0 --> 2 

  Char. 383: 0 --> 1 

  Char. 385: 0 --> 1 

  Char. 394: 0 --> 1 

  Char. 400: 0 --> 1 

  Char. 440: 1 --> 0 

  Char. 445: 1 --> 0 

  Char. 454: 0 --> 1 

Node 92 (Camarasaurus)

  Char. 91: 1 --> 0 

  Char. 332: 0 --> 1 

  Char. 390: 0 --> 1 

  Char. 407: 1 --> 0 

Node 93 

  Char. 364: 0 --> 1 

Node 94 

  Char. 389: 1 --> 0 

Node 95 (Dicraeosauridae)

  Char. 430: 0 --> 1 

  Char. 442: 0 --> 1 

  Char. 451: 0 --> 1 

  Char. 460: 1 --> 0 

  Char. 475: 1 --> 0 

Node 96 (Flagellicaudata)

  Char. 7: 0 --> 1 

  Char. 14: 0 --> 1 

  Char. 50: 1 --> 0 

  Char. 53: 0 --> 1 

  Char. 55: 0 --> 1 

  Char. 57: 0 --> 1 

  Char. 58: 0 --> 1 

  Char. 86: 0 --> 1 

  Char. 103: 0 --> 1 

  Char. 121: 0 --> 1 

  Char. 122: 1 --> 0 

  Char. 125: 0 --> 1 

  Char. 178: 0 --> 1 

  Char. 201: 1 --> 0 

  Char. 249: 0 --> 1 

  Char. 260: 0 --> 1 

  Char. 275: 1 --> 0 

  Char. 302: 0 --> 1 

  Char. 303: 0 --> 1 

  Char. 304: 0 --> 1 

  Char. 332: 0 --> 1 

  Char. 351: 1 --> 0 

  Char. 370: 0 --> 1 

  Char. 422: 0 --> 1 

  Char. 423: 1 --> 0 

  Char. 424: 0 --> 1 

  Char. 462: 0 --> 1 

  Char. 464: 0 --> 1 

Node 97 (Diplodocimorpha)

  Char. 281: 0 --> 1 

  Char. 299: 0 --> 1 

  Char. 316: 0 --> 1 

  Char. 317: 0 --> 1 

  Char. 408: 0 --> 1 

Node 98 (Diplodocoidea)

  Char. 213: 0 --> 1 

  Char. 214: 0 --> 1 

  Char. 427: 1 --> 0 

  Char. 453: 0 --> 1 

Node 99 (Neosauropoda)

  Char. 224: 0 --> 1 

  Char. 351: 0 --> 1 

  Char. 382: 0 --> 1 

  Char. 457: 0 --> 1 

  Char. 460: 0 --> 1 

Node 100 

  Char. 137: 1 --> 0 

  Char. 170: 2 --> 1 

  Char. 230: 0 --> 1 

  Char. 240: 0 --> 1 

  Char. 262: 0 --> 1 

Node 101 (Brachiosauridae)

  Char. 132: 0 --> 1 

  Char. 182: 0 --> 1 

  Char. 263: 0 --> 1 

  Char. 266: 01 --> 2 

  Char. 401: 1 --> 2 

  Char. 435: 0 --> 1 

Node 102 (Titanosauriformes)

  Char. 129: 1 --> 2 

  Char. 352: 0 --> 1 

  Char. 367: 1 --> 0 

  Char. 386: 1 --> 0 

  Char. 414: 0 --> 1 

  Char. 428: 0 --> 1 

  Char. 429: 1 --> 2 

Node 103 

  Char. 130: 1 --> 0 

  Char. 135: 0 --> 1 

  Char. 171: 0 --> 1 

Node 104 

  Char. 175: 0 --> 1 

  Char. 249: 1 --> 0 

  Char. 250: 0 --> 1 

  Char. 283: 0 --> 1 

Node 105 

  Char. 411: 0 --> 1 

  Char. 419: 1 --> 0 

  Char. 447: 0 --> 1 

Node 106 

  Char. 306: 0 --> 1 

  Char. 331: 0 --> 1 

Node 107 (Diplodocinae)

  Char. 266: 0 --> 1 

  Char. 272: 0 --> 1 

  Char. 299: 1 --> 3 

  Char. 420: 0 --> 1 

  Char. 441: 0 --> 1 

  Char. 458: 0 --> 1 

Node 108 (Diplodocidae)

  Char. 16: 0 --> 1 

  Char. 22: 0 --> 1 

  Char. 24: 0 --> 1 

  Char. 49: 0 --> 1 

  Char. 126: 0 --> 1 

  Char. 198: 0 --> 1 

  Char. 207: 0 --> 1 

  Char. 223: 1 --> 2 

  Char. 257: 0 --> 1 

  Char. 258: 0 --> 1 

  Char. 262: 1 --> 0 

  Char. 313: 0 --> 1 

  Char. 315: 0 --> 1 

  Char. 318: 0 --> 1 

  Char. 320: 0 --> 1 

  Char. 382: 1 --> 0 

  Char. 391: 0 --> 1 

  Char. 427: 0 --> 1 

Node 109 (Somphospondyli)

  Char. 184: 1 --> 0 

  Char. 185: 0 --> 1 

  Char. 188: 1 --> 0 

  Char. 227: 1 --> 2 

  Char. 232: 0 --> 1 

  Char. 262: 1 --> 0 

  Char. 273: 1 --> 0 

  Char. 285: 1 --> 2 

  Char. 365: 0 --> 1 

  Char. 366: 1 --> 0 

  Char. 370: 0 --> 1 

  Char. 406: 0 --> 1 

Node 110 (Limaysaurinae)

  Char. 184: 1 --> 0 

  Char. 318: 0 --> 1 

  Char. 363: 0 --> 1 

Node 111 

  Char. 335: 0 --> 1 

  Char. 416: 0 --> 1 

  Char. 418: 0 --> 1 

  Char. 420: 0 --> 1 

Node 112 (Rebbachisauridae)

  Char. 188: 1 --> 0 

  Char. 196: 0 --> 1 

  Char. 324: 0 --> 1 

  Char. 333: 0 --> 2 

  Char. 417: 0 --> 1 

  Char. 459: 0 --> 1 

  Char. 461: 0 --> 1 

Node 113 (Nigersaurinae)

  Char. 261: 0 --> 1 

  Char. 291: 0 --> 1 

  Char. 434: 0 --> 1 

Node 114 

  Char. 279: 1 --> 0 

  Char. 429: 1 --> 0 

Node 115 

  Char. 162: 0 --> 1 

  Char. 183: 1 --> 0 

  Char. 287: 0 --> 1 

  Char. 292: 0 --> 1 

  Char. 349: 0 --> 1 

  Char. 450: 0 --> 1 

Node 116 

  Char. 222: 0 --> 1 

  Char. 258: 1 --> 0 

Node 117 

  Char. 137: 0 --> 1 

  Char. 158: 0 --> 1 

  Char. 173: 0 --> 1 

  Char. 178: 1 --> 0 

  Char. 224: 1 --> 0 

  Char. 237: 0 --> 1 

  Char. 249: 1 --> 0 

  Char. 253: 1 --> 0 

  Char. 295: 0 --> 1 

Node 118 (Apatosaurinae)

  Char. 134: 0 --> 1 

  Char. 185: 0 --> 1 

  Char. 200: 0 --> 1 

  Char. 215: 0 --> 1 

  Char. 219: 0 --> 1 

  Char. 279: 0 --> 1 

  Char. 353: 0 --> 1 

  Char. 371: 0 --> 1 

Node 119 

  Char. 133: 1 --> 0 

  Char. 167: 1 --> 2 

  Char. 178: 1 --> 0 

  Char. 254: 0 --> 1 

  Char. 267: 1 --> 0 

  Char. 280: 0 --> 1 

Node 120 

  Char. 129: 1 --> 0 

  Char. 131: 1 --> 0 

  Char. 169: 0 --> 1 

  Char. 176: 0 --> 1 

  Char. 224: 1 --> 0 

  Char. 226: 1 --> 0 

  Char. 227: 1 --> 0 

  Char. 245: 0 --> 1 

  Char. 251: 1 --> 0 

Node 121 

  Char. 205: 0 --> 1 

  Char. 252: 0 --> 1 

  Char. 259: 0 --> 1 

  Char. 269: 0 --> 1 

  Char. 292: 1 --> 0 

  Char. 327: 1 --> 0 

  Char. 328: 1 --> 0 

  Char. 360: 1 --> 0 

  Char. 364: 0 --> 1 

  Char. 368: 0 --> 1 

Node 122 

  Char. 162: 1 --> 0 

  Char. 258: 0 --> 1 

  Char. 263: 0 --> 1 

  Char. 389: 0 --> 1 

Node 123 

  Char. 273: 1 --> 0 

  Char. 387: 0 --> 1 

Node 124 

  Char. 270: 0 --> 1 

  Char. 272: 0 --> 1 

Node 125 

  Char. 266: 0 --> 1 

  Char. 437: 0 --> 1 

Node 126 

  Char. 221: 0 --> 1 

  Char. 328: 1 --> 0 

Node 127

  Char. 207: 1 --> 0 

  Char. 217: 0 --> 1 

Node 128 (Brontosaurus excelsus)

  Char. 309: 0 --> 1 

Node 129 

  Char. 127: 1 --> 2 

  Char. 167: 1 --> 0 

  Char. 187: 0 --> 1 

  Char. 236: 0 --> 1 

  Char. 425: 0 --> 1 

Node 130 (Galeamopus hayi)

  Char. 165: 1 --> 0 

Node 131 

  Char. 34: 0 --> 1 

  Char. 59: 1 --> 2 

  Char. 66: 0 --> 1 

  Char. 78: 1 --> 0 

  Char. 89: 0 --> 1 

Node 132 

  Char. 11: 0 --> 1 

Node 133 (Galeamopus)

  Char. 61: 1 --> 0 

  Char. 145: 0 --> 1 

  Char. 147: 0 --> 1 

  Char. 150: 0 --> 1 

Node 134 

  Char. 297: 0 --> 1 

Node 135 

  Char. 314: 0 --> 1 

Node 136 

  Char. 323: 0 --> 1 

  Char. 443: 1 --> 0 

  Char. 444: 0 --> 1 

  Char. 454: 0 --> 1 

Node 137 (Supersaurus vivianae)

  Char. 183: 1 --> 2 

Node 138 (Kaatedocus)

  Char. 16: 1 --> 0 

  Char. 26: 1 --> 0 

  Char. 31: 0 --> 1 

  Char. 79: 1 --> 0 

  Char. 84: 2 --> 1 

  Char. 130: 1 --> 0 

  Char. 165: 2 --> 1 

  Char. 177: 0 --> 1 

  Char. 186: 1 --> 0 

  Char. 201: 0 --> 1 

  Char. 202: 1 --> 0 

  Char. 204: 1 --> 0 

  Char. 210: 0 --> 1 

  Char. 211: 0 --> 1 

  Char. 212: 1 --> 0 

  Char. 213: 1 --> 0 

Node 139 

  Char. 65: 0 --> 1 

  Char. 95: 0 --> 1 

  Char. 156: 1 --> 0 

  Char. 163: 0 --> 1 

  Char. 165: 1 --> 2 

Node 140 

  Char. 133: 1 --> 0 

  Char. 139: 1 --> 2 

  Char. 183: 1 --> 0 

  Char. 186: 0 --> 1 

Node 141 

  Char. 153: 1 --> 0 

  Char. 195: 0 --> 1 

  Char. 237: 0 --> 1 

  Char. 257: 1 --> 2 

  Char. 268: 0 --> 1 

  Char. 280: 0 --> 1 

  Char. 332: 1 --> 0 

  Char. 334: 0 --> 1 

  Char. 366: 1 --> 0 

  Char. 380: 0 --> 1 

  Char. 439: 0 --> 1 

  Char. 460: 1 --> 0 

Node 142 (Tornieria)

  Char. 378: 0 --> 1 

  Char. 417: 0 --> 1 

  Char. 425: 0 --> 1 

Node 143 (DiCa)

  Char. 138: 0 --> 1 

  Char. 246: 0 --> 1 

  Char. 276: 0 --> 1 

  Char. 294: 1 --> 0 

  Char. 420: 1 --> 0 

Node 144 (Diplodocus)

  Char. 181: 1 --> 0 

  Char. 264: 1 --> 0 

  Char. 279: 0 --> 1 

  Char. 299: 3 --> 4 

  Char. 307: 0 --> 1 

  Char. 330: 0 --> 1 

  Char. 335: 0 --> 1 

  Char. 467: 0 --> 1 

Node 145 (Diplodocus hallorum)

  Char. 233: 0 --> 1 

  Char. 336: 0 --> 1 

  Char. 339: 0 --> 1 

  Char. 342: 0 --> 1 

  Char. 356: 0 --> 1 

Node 146 

  Char. 257: 2 --> 1 

Node 147 

  Char. 425: 0 --> 1 

Node 148 

  Char. 457: 0 --> 1 

  Char. 464: 0 --> 1 

  Char. 470: 0 --> 1 

Node 149 

  Char. 86: 0 --> 1
